# Differences in Reproductive Behavior between Spawning and Non-Spawning Zebrafish Pairs and the Effects of 17α-Ethinylestradiol (EE2)

**DOI:** 10.3390/toxics4030022

**Published:** 2016-09-06

**Authors:** Per G. Henriksen, Kristian Beedholm, Erik Baatrup

**Affiliations:** Department of Bioscience, Zoophysiology, C.F. Moellers Alle 3, Aarhus University, building 1131, DK-8000 Aarhus C, Denmark; per.henriksen@bios.au.dk (P.G.H.); kristian.beedholm@bios.au.dk (K.B.)

**Keywords:** zebrafish, reproductive behavior, spawning, 17α-ethinylestradiol, EE2

## Abstract

Reproductive success manifested by spawning and fertilization, in most fish, depends partly on an appropriate courtship behavior by both sexes. The zebrafish reproductive behavior can be resolved in some of its constituent elements by a computerized vision system and described in unbiased quantitative terms. Pairs of adult male and female zebrafish were monitored with automatic video tracking at 16 Hz for 45 min in a tank with a spawning area in one corner. Subsequently, spawning, if any, was registered and the swimming behavior and mutual interactions of the two fish were quantified. Further, temporal and frequency distributions of average velocity and turning rate were produced. It is demonstrated that the courtship behavior in spawning pairs differs markedly from non-spawning pairs with differences in both male and female behavior. EE2 (17α-ethinylestradiol), a contraceptive hormone found in aquatic environments, has only a slight effect on these behavior differences between spawning and non-spawning pairs.

## 1. Introduction

Reproductive success in fish depends partly on the ability to perform correct courtship behavior. The zebrafish reproductive behavior has been outlined by Darrow and Harris [1] and Hutter et al. [2], and the entire known zebrafish behavioral repertoire by Kalueff et al. [3]. Besides visual and tactile stimuli, pheromones also play a crucial role in mating behavior [4,5,6]. The evident relationship between zebrafish sexual behavior and reproductive success was demonstrated by Larsen et al. [7], where significant differences were demonstrated in eight components of the male zebrafish courtship behavior of individuals inducing spawning and those not inducing spawning, respectively. The study by Larsen et al. [7] focused solely on the male reproductive behavior. In the present investigation, we compare the courtship behavior of both sexes in zebrafish pairs with successful and unsuccessful mating trials, respectively. Also, we further include novel measures of zebrafish swimming and mating behavior.

A second objective of the present investigation was to further uncover the effect of the contraceptive estrogenic pharmaceutical 17α-ethinylestradiol (EE2) on the zebrafish reproductive behavior. In Europe, EE2 is present in effluents and surface waters at concentrations from below the detection limit of around 0.5 ng·L^−1^ and up to 15 ng·L^−1^, with the majority of detections below 5 ng·L^−1^ [8,9,10,11,12]. In other parts of the world, higher concentrations of EE2 have been detected, for instance 42 ng·L^−1^ in a Canadian sewage treatment effluent [10] and 831 ng·L^−1^ in an American stream (maximum of 139 sampling sites) [13]. EE2 is reported to disturb several crucial processes in fish, even at very low-nanogram concentrations. Among many physiological and morphological effects, EE2 is known to reduce fecundity in fathead minnow and zebrafish [14,15,16]. We have previously demonstrated that prolonged exposure to low-nanoscale concentrations (<5 ng·L^−1^) of EE2 only has a limited effect on the male zebrafish courtship behavior [7]. In contrast, the reproductive behavior of unexposed female zebrafish paired with EE2-exposed males is significantly altered when compared with females paired with unexposed males [17]. It has previously been demonstrated that EE2 reduces male-to-male aggression and male-to-female association [18]. Here, we quantitatively compare 11 components of the reproductive behavior in spawning and non-spawning zebrafish pairs where some males were exposed to 1.26 ng·EE2·L^−1^.

## 2. Results

The mortality was extremely low when registered from 47 days post fertilization (dpf) (time of the down-scaling to about 60 individuals in each tank) and until the fish were sampled for experimentation between 111 and 177 dpf. Only two fish died in the control tanks and one fish in the exposure tanks. The sex ratio of all the fish in the control and exposure tanks showed that the EE2-treatment significantly reduced the percentage of phenotypic males from 56% to 26%. Of the total 168 test fish that were each paired with a standard female for behavior measurements, 150 turned out to be phenotypic males with distinct testes. Only these 150 cases were included in the subsequent behavior analyses. Hereof, 89 were control males (not exposed to EE2) while 61 males were exposed to 1.26 ng·EE2·L^−1^. In the 89 pairs with control males, 30 pairs were spawning (34%), whereas only 14 of the 61 EE2-exposed males (23%) induced spawning, demonstrating a trend of suppressed spawning by EE2 (Mann–Whitney U, *p* = 0.076).

### 2.1. The Reproductive Behavior in Spawning and Non-Spawning Zebrafish Pairs

The reproductive behavior varied notably between spawning and non-spawning pairs, where both males and females demonstrated significant differences in their swimming patterns and mutual interactions.

In the group of zebrafish pairs with unexposed (control) males (Table 1), both sexes were swimming significantly longer distances when the courtship resulted in spawning than in non-spawning pairs. For both sexes, this was caused by a higher average swimming velocity. Also, both sexes had a higher turning rate in spawning pairs, but there was no preference (turn bias) for right or left turning in either sex. Further, males and females spent significantly longer times and were swimming significantly longer distances in the spawning area in spawning pairs. Males of spawning pairs also tended to make more visits to the spawning area. In all three behavioral parameters relating the proximity of male and female, including increased number of meetings, increased total time of meetings and a lower average distance between male and female in spawning pairs were highly significant when compared with non-spawning pairs (Table 1).

### 2.2. Effect of 17α-Ethinylestradiol (EE2)

The concentrations of EE2 in the nominally 3 ng EE2·L^−1^ exposure tanks were measured to be only 1.26 ng·L^−1^ (42% of the nominal concentration), implying that the fish were exposed to a markedly lower concentration than intended. In the zebrafish pairs, where the male grew up under EE2 exposure (Table 2), the differences in courtship behavior between spawning and non-spawning pairs varied from the unexposed pairs. In the males inducing spawning, the path length was significantly higher, whereas the maximum velocity was significantly lower, both compared to males in non-spawning pairs and to all males in the unexposed zebrafish. Also, there were a significantly lower number of visits to the spawning area and swimming distance of males in the non-spawning pairs compared with males in spawning pairs (Table 2). The differences in female behavior in spawning and non-spawning were almost identical to females in the pairs with unexposed males, with the exception that non-spawning females paired with exposed males entered the spawning area significantly fewer times than the spawning females.

The effects of EE2 on the zebrafish reproductive behavior were further investigated in spawning and non-spawning pairs by comparing the 11 measured behavioral parameters in pairs where the male had been subjected to 0 or 1.26 ng·EE2·L^−1^, respectively. The behavioral differences were most pronounced in the non-spawning pairs (Table 3), and surprisingly only in the unexposed female partners. Here, the females, paired with exposed males, swam significantly shorter distances with markedly lower average velocities when compared with females paired with unexposed males. Also, these females had significantly fewer visits to the spawning area, were swimming shorted distances within this area, and had noticeably fewer meetings with the male. In the spawning pairs, only the average velocity of the female and the number of meetings differed significantly between pairs with exposed and control males, respectively. 

### 2.3. Temporal and Frequency Distributions

**Figure 1:**
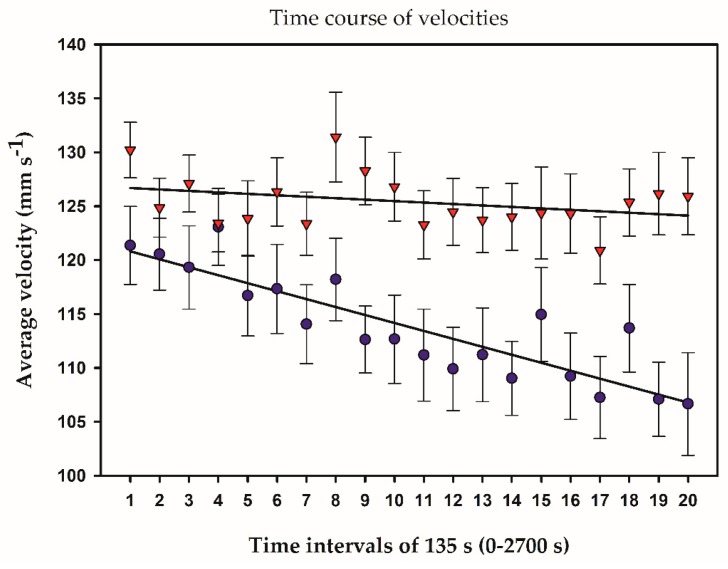
Average velocity of unexposed male zebrafish in spawning (-▼-) and non-spawning (-●-) pairs during the 45 min recording. Average velocities were categorized into 20 intervals of 135 s. As evident from the regression lines, males in spawning pairs maintained a nearly constant average velocity, whereas males in non-spawning pairs gradually reduced their average velocity. Error bars represent SEM.

During the 45 min of recording zebrafish courtship behavior, the average velocities of the two fish were each categorized into 20 time intervals of 135 s. Average swimming velocity of unexposed males in spawning pairs was constant during the entire 45 min observation period, differing significantly (χ^2^ = 119, df = 20, *p* < 0.0001) from the males in non-spawning pairs, which gradually decreased their average velocity (Figure 1).

In contrast, female average velocity of spawning pairs (data not shown) was statistically similar to non-spawning pairs during the recording period (χ^2^ = 20, df = 20, *p* = 0.5000). EE2 at 1.26 ng·L^−1^ had no effect on male average swimming velocity over time (χ^2^ = 26, df = 20, *p* = 0.1500). Spawning females paired with EE2-exposed males had similar average velocities over time compared with females paired with unexposed males (χ^2^ = 22, df = 20, *p* = 0.2500). In contrast, non-spawning females paired with EE2-exposed males had significantly lower average velocities over time than females paired with unexposed males (χ^2^ = 59, df = 20, *p* < 0.005).

**Figure 2:**
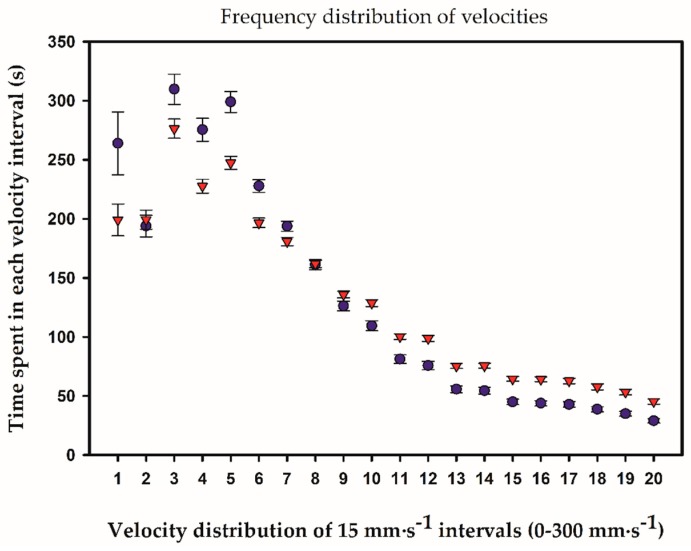
Velocity distribution of unexposed male zebrafish in spawning (▼) and non-spawning (●) pairs during the 45 min recording. Velocities from 0 mm·s^−1^ to 300 mm·s^−1^ were categorized into 20 intervals of 15 mm·s^−1^. Males in spawning pairs spent less time at lower velocities and more time at higher velocities compared with males in non-spawning pairs. Note the diminutive error bars (SEM), demonstrating the uniform administration of swimming velocity among male zebrafish. Error bars represent SEM.

The velocity frequency distributions of the male and female zebrafish were categorized into 20 bins of 15 mm·s^−1^, ranging from 0 to 300 mm·s^−1^. Males inducing spawning used less time at velocities below 120 mm·s^−1^ and consequently more time at higher velocities than males in non-spawning pairs (χ^2^ = 119, df = 20, *p* < 0.0001) (Figure 2). Females had similar velocity distributions in spawning and non-spawning pairs (χ^2^ = 22, df = 20, *p* = 0.250). EE2 had a statistically significant effect on the male velocity distribution in non-spawning pairs (χ^2^ = 47, df = 20, *p* < 0.005), but this result can be explained as a byproduct of unexposed males in spawning pairs spending more time in all velocity intervals. In spawning pairs, unexposed and EE2-exposed males had very similar velocity distributions (χ^2^ = 13, df = 20, *p* = 0.900).

**Figure 3:**
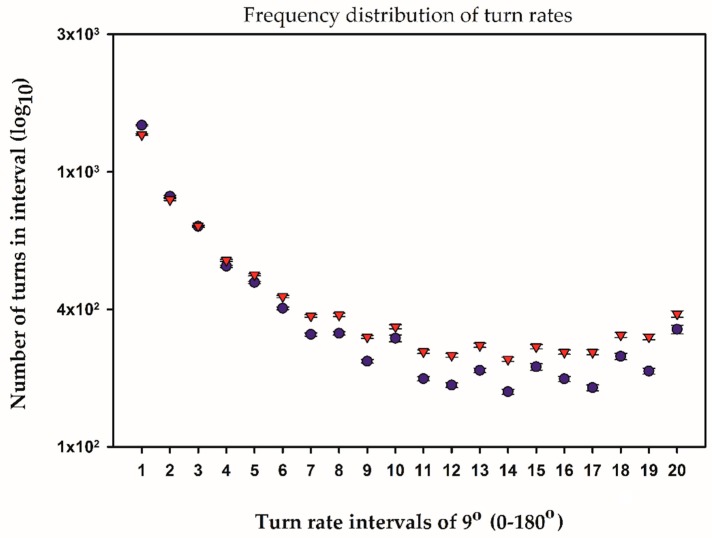
Average turn rate of unexposed male zebrafish in spawning (▼) and non-spawning (●) pairs during the 45 min recording. Turn rates were categorized into 20 intervals of 9°, from 0° to 180°. The males in spawning pairs made more turns above 45° than males in non-spawning pairs. The high number of turns arises because of the 16 Hz sampling rate, producing 43.200 angles. Note the logarithmic *Y*-axis. Note also the diminutive error bars (SEM), demonstrating the very uniform turning behavior in male zebrafish.

The turn rate (degrees turned for each recorded position) was categorized into 20 intervals of 9°, from 0° to 180°. The unexposed males in spawning pairs made significantly more turns above 45° than males in non-spawning pairs (χ^2^ = 1039, df = 20, *p* < 0.0001) (Figure 3). This was also true for the spawning females (χ^2^ = 97, df = 20, *p* < 0.0001), having a higher turn rate than females in non-spawning pairs. EE2 had no effect on male (χ^2^ = 15, df = 20, *p* = 750) or female (χ^2^ = 22, df = 20, *p* = 0.250) turning rate. Finally, there were no differences in turn bias (difference between right and left turning) in any of the calculated comparisons.

## 3. Discussion

The present study provides the first quantitative documentation of differences in both male and female zebrafish reproductive behavior during successful and unsuccessful courtship, respectively. The experimental setup has previously been adopted in effect studies of endocrine disruptors on the zebrafish reproductive behavior [7,17,19,20]. Here, we further extended the analyses to include frequency distributions and temporal progress of measured endpoints during the course of the fish behavior trials. During recording, an image of the two fish was captured 16 times per second (16 Hz). This assures acquisition of positions for each fish body length during swimming, limiting the underestimation of the true swimming path and turn rate. 

The zebrafish obviously considered the plastic trays with glass beads as suitable spawning grounds, since the male incessantly enticed the female to follow him to the tray and because eggs were laid here. The male successfully courted the female in 34% of the cases, resulting in a spawning success rate markedly lower than the 50%–70% reported in other laboratory studies [1,5]. Possible reasons could be the restricted recording period of 45 min, the limited space available in the test tanks, lack of plants, and because small males were selected in order to facilitate the discrimination between the male and the female in the digitized image. These deviations from a natural courtship environment may well underestimate the spawning potential and in particular the effects of EE2. The dominance rank of the males may also be in play although Spence and Smith [21] found that female zebrafish does not favor dominant males in the absence of male competition.

The male courtship behavior in spawning pairs differed significantly from males of non-spawning pairs in 8 of the 11 behavioral components that were considered for each sex. In spawning pairs, the male swam longer and faster, made more turns, spent more time and swam longer distances in the spawning area, made more contacts and spent more time close to female and overall kept a much shorter distance to her. Only maximum swimming velocity, turn bias (imbalance between left and right turning), and number of visits to spawning area were statistically similar in the two groups.

During the 45 min of recording courtship behavior, the average swimming velocity of males in spawning pairs was nearly constant as opposed to males in non-spawning pairs, which gradually reduced their swimming velocity. Also, males of spawning pairs spent significantly more time at higher velocities and less time at lower velocities than males in non-spawning pairs. Although speculative, it appears as if proper courtship and the forthcoming mating success were recognized by the males in spawning pairs right from the start. This was probably brought about by female encouraging responses mediated by visual, mechanical, or chemical stimuli [6,22], signals inaccessible to our vision system. 

An elevated turn rate was demonstrated in both males and females of spawning pairs, suggesting a more varied or changing swimming pattern than in non-spawning pairs. A similar higher turn rate in spawning pairs was also noted by Larsen et al. [7].

Females in spawning pairs swam longer and had a higher turning rate than the females in non-spawning pairs. We interpret this as a response to the male´s approaches, expressed by the higher number of meetings, more time in proximity, and a shorter average distance of the sexes in spawning pairs. Spawning females also spent more time and swam longer distances in the spawning area than females in non-spawning pairs. We expected more female visits to the spawning area in spawning pairs, which was also the trend, but not statistically significant.

Previous studies have demonstrated that spawning activity [18] and breeding success [23] are not negatively affected by 17α-ethinylestradiol in zebrafish. Also, in a similar study on medaka (*Oryzias melastigma*), Lee et al. [24] found that EE2 had no influence on swimming performance (up to 50 ng·L^−1^) or reproductive behaviors, including following, dancing, and copulation at 10 ng·L^−1^. At higher concentrations (50–100 ng·L^−1^), dancing, copulation, and spawning were significantly suppressed [24]. In fathead minnows (*Pimephales promelas*) exposed to 2 or 8 ng·L^−1^ EE2, Majewski et al. [25] found an impaired male ability to compete and acquire territories. A similar result was obtained with fathead minnows exposed to a much higher concentration of EE2, 40 ng·L^−1^, for 21 days, also demonstrating a decreased ability of exposed males to compete with control males for spawning substrate as well as chasing male competitors [26]. Suppressed male sex characteristics and reduced egg fertilization success were demonstrated in fathead minnows after prolonged exposure to 3.5, 9.6, and 23 ng·L^−1^ EE2 [27]. EE2 also influenced reproduction in the sand goby (*Pomatoschistus minutus*). Delayed nest-building and suppressed courtship and leading behaviors were demonstrated in male sand gobies exposed to 11 ng·L^−1^ for 1–4 weeks [28]. These exposed males were also significantly less aggressive than control males [28]. Saaristo and coworkers also revealed disrupted sexual selection in sand gobies exposed to EE2 at 5 and 24 ng·L^−1^ [29], and altered fanning behavior during both courtship and parental care when exposed to 41 ng·L^−1^ EE2 for 10–31 days [30].

In the present study, prolonged EE2 exposure to 1.26 ng·L^−1^ had an influence on zebrafish courtship behavior and the differences between spawning and non-spawning pairs. When comparing the unexposed male courtship behavior in spawning pairs with the similar EE2-exposed male (data not shown), no statistical differences in the behavior elements considered here were found. This was also the case with non-spawning pairs, except that the EE2-exposed males made significantly fewer visits to the spawning area. Females in spawning pairs behaved similarly towards males, irrespective of exposure. Females in non-spawning pairs, on the other hand, responded to exposed males with shorter swimming distances and lower average velocities, in agreement with previous findings [17]. Of course, the argument can rightly be turned around, namely that some EE2-exposed males were unable to stimulate the females properly, resulting in an unsuccessful reproduction. The limited effect of EE2 on the courtship behavior elements addressed in this study is in accordance with Larsen et al. [7] demonstrating that courtship behavior of male zebrafish was nearly unaffected by EE2 up to 5 ng·L^−1^, although their secondary sexual characteristics were altered down to 0.05 ng·L^−1^ [7]. In contrast, the female courtship behavior was significantly altered [17] when paired with EE2-exposed males, despite the fact that these females had never been exposed to EE2. Thus, although most addressed elements of male behavior are unaltered by EE2, the overall female impression of the male leads to a significant altered female behavior. The present study supplements these findings, demonstrating that both male and female mating behaviors are decisive in successful reproduction. The effects of EE2 on the zebrafish reproductive behavior demonstrated in this laboratory study may have more far-reaching and serious consequences at the population level. In an EE2-contaminated environment, a suppressed male attraction may well intimidate reproduction, progeny survival, and consequently, population stability.

## 4. Materials and Methods

The present study extracted new data and performed new analyses on the zebrafish used in Baatrup and Henriksen [17]. For detailed information on fish handling, exposure and behavioral measurements, please consult Baatrup and Henriksen [17].

Briefly, zebrafish (*Danio rerio*) were raised in continuous flow-through tanks from eggs to sexual maturity with one exposure group of three replicates receiving 3 ng·L^−1^ nominal concentration of 17α-ethinylestradiol (EE2) and a control group of three replicates receiving only the carrier solvent, acetone. The actual concentration of 1.26 ng·L^−1^ EE2 in the water was quantified using high-performance liquid chromatography tandem mass spectrometry (HPLC—MS/MS) [17]. During the period from 111 to 177 days post fertilization (dpf), behavioral trials were carried out with 168 male-looking test fish from the exposure and control tanks, respectively. Temporarily, the measurements were equally distributed between control and exposed fish. Later, after dissection, the trials including phenotypic males could be identified. Courtship behavior was quantified using one presumed male and one “standard female”. “Standard females” were large mature females from the stock tank, which were ready to spawn at the behavioral trial, being isolated from males for at least five days prior to experimentation [5,31]. The behavior test tanks (28 × 21 × 13 cm) contained 6 cm stock tank water (2.2 L) at 25 °C and a plastic tray (10 × 10 cm) with transparent 5 mm diameter glass beads, placed in one corner, serving as an artificial spawning ground. The test tank was placed on a sheet of glass 50 cm above diffusely lit white paper (79 lux). When viewed from above by the camera, this arrangement resulted in clear silhouettes of the two fish, where the male was distinguishable from the larger female. The digital video signal from the camera consisted of a 1024 × 768 pixel image, giving a 0.27 mm spatial resolution of the visual field. The behavioral measurements were controlled by the MOTIO vision system (Department of Bioscience, University of Aarhus, Aarhus, Denmark). During the 45 min recording, the images were captured at 16 frames per second (16 Hz). The male and female courtship behavior was evaluated on the basis of the 11 parameters listed in Table 1 and Table 2. Additionally, frequency distributions (20 intervals) of swimming velocities between 0 and 300 mm·s^−1^ were extracted from the data file, together with average swimming velocity for each 135 s during the 45 min (2700 s) recording. Likewise, frequency distributions of turn rate (number of turns in each interval from 0° to 180°) were calculated. After the 45 min behavioral measurement, the female was returned to the stock aquarium and the test fish was processed for gender determination. The test tank was then inspected for eggs demonstrating whether spawning had taken place.

The three replicates in each of the two groups were analyzed for normality and homogeneity of variances for all the 11 behavioral parameters. When complying, differences in mean value were analyzed with Analysis of Variance ANOVA, and the three replicates were subsequently pooled. Differences in behavior components were detected using independent-samples *T*-Test. If data did not meet homogeneity of variances, the Welch test was used. In the few cases, where data did not comply with normality, but where simple transformation resulted in normality, the *T*-Test was carried out. The χ^2^-test was used for comparison of frequency distributions, presuming that the period between data points precluded correlation. Spawning frequencies were compared using the Mann–Whitney *U* test. All statistical tests were performed in SPSS 22 for Windows (IBM Corporation, New York, NY, USA), except the χ^2^-tests which were performed in Excel (Microsoft Office 10). Data are presented as mean value ± S.E.M. and a significance level of 0.05.

## Figures and Tables

**Table 1:** Differences in behavior in spawning and non-spawning control zebrafish pairs.

Behavior Components	−Spawning (*n* = 59)	+Spawning (*n* = 30)	*p*-Value
Total path (m) Males Total path (m) Females	304 ± 7.46 209 ± 8.03	339 ± 8.12 259 ± 8.52	**0.005** **<0.001**
Max velocity (mm·s^−1^) Males Max velocity (mm·s^−1^) Females	738 ± 21.5 537 ± 23.9	709 ± 23.7 466 ± 29.2	0.403 0.077
Average velocity (mm·s^−1^) Males Average velocity (mm·s^−1^) Females	117 ± 2.72 87 ± 2.59	127 ± 3.30 100 ± 2.97	**0.017** **0.009**
Turn rate·s^−1^ (degrees) Males Turn rate·s^−1^ (degrees) Females	407 ± 10.0 444 ± 13.6	542 ± 18.6 507 ± 21.7	**<0.001** **0.012**
Turn bias·s^−1^ (degrees) Males Turn bias·s^−1^ (degrees) Females	−1.23 ± 1.01 −1.59 ± 1.37	−3.39 ± 1.59 −1.18 ± 1.96	0.237 0.863
Time in spawning area (s) Males Time in spawning area (s) Females	544 ± 28.6 430 ± 34.7	762 ± 75.7 669 ± 74.3	**0.002** **0.001**
Path in spawning area M (m) Males Path in spawning area F (m) Females	59.5 ± 2.76 42.0 ± 2.44	88.2 ± 7.89 68.4 ± 6.90	**<0.001** **<0.001**
Visits to spawning area Males Visits to spawning area Females	340 ± 14.3 241 ± 13.2	390 ± 27.2 286 ± 24.4	0.074 0.082
Number of contacts between male and female	1026 ± 55.3	1711 ± 85.5	**<0.001**
Total time (s) of contact between male and female	454 ± 36.6	746 ± 69.2	**<0.001**
Average distance (mm) between male and female	73.4 ± 2.52	47.7 ± 3.28	**<0.001**

All entries are average values ± Standard Error of the Mean (SEM). Values that are significantly different (*p* < 0.05) are emphasized in bold. In the “Turn bias” rows, negative values indicate a bias towards right turning.

**Table 2:** Differences in behavior in spawning and non-spawning zebrafish pairs where the males were exposed to 1.26 ng·EE2·L^−1^.

Behavior Components	−Spawning (*n* = 47)	+Spawning (*n* = 14)	*p*-Value
Total path (m) Males Total path (m) Females	293 ± 8.26 181 ± 8.92	335 ± 7.41 252 ± 7.53	**0.009** **<0.001**
Max velocity (mm·s^−1^) Males Max velocity (mm·s^−1^) Females	742 ± 19.8 397 ± 19.0	632 ± 38.7 415 ± 51.4	**0.011** 0.700
Average velocity (mm·s^−1^) Males Average velocity (mm·s^−1^) Females	115 ± 3.19 79.3 ± 3.10	125 ± 2.80 96.4 ± 1.67	0.098 **0.005**
Turn rate·s^−1^ (degrees) Males Turn rate·s^−1^ (degrees) Females	423 ± 11.1 437 ± 16.6	555 ± 30.1 487 ± 28.0	**<0.001** 0.145
Turn bias·s^−1^ (degrees) Males Turn bias·s^−1^ (degrees) Females	−3.23 ± 1.35 −1.98 ± 1.30	−0.44 ± 3.64 1.61 ± 2.67	0.380 0.203
Time in spawning area (s) Males Time in spawning area (s) Females	544 ± 46.1 436 ± 56.1	791 ± 88.7 639 ± 82.4	**0.014** 0.076
Path in spawning area M (m) Males Path in spawning area F (m) Females	58.7 ± 4.72 35.3 ± 4.04	92.7 ± 8.96 65.2 ± 9.08	**0.001** **0.006**
Visits to spawning area Males Visits to spawning area Females	304 ± 13.6 189 ± 11.9	387 ± 19.3 276 ± 23.1	**0.003** **0.001**
Number of contacts between male and female	959 ± 72.8	1816 ± 128	**<0.001**
Total time (s) of contact between male and female	466 ± 44.8	785 ± 85.9	**0.001**
Average distance (mm) between male and female	74.6 ± 3.04	44.5 ± 4.39	**<0.001**

All entries are average values ± SEM. Values that are significantly different (*p* < 0.05) are emphasized in bold. In the “Turn bias” row, negative values indicate a bias towards right turning while the positive value indicates a bias towards left turning.

**Table 3:** Differences in reproductive behavior in non-spawning zebrafish pairs where the males were exposed to 0 and 1.26 ng·EE2·L^−1^, respectively.

Behavior Components	0 ng·L^−1^ (*n* = 59)	1.26 ng·L^−1^ (*n* = 47)	*p*-Value
Total path (m) Males Total path (m) Females	304 ± 7 256 ± 13	293 ± 8 194 ± 10	0.329 **<0.001**
Max velocity (mm·s^−1^) Males Max velocity (mm·s^−1^) Females	738 ± 22 1179 ± 72	742 ± 20 779 ± 67	0.901 **<0.001**
Average velocity (mm·s^−1^) Males Average velocity (mm·s^−1^) Females	113 ± 3 105 ± 4	110 ± 3 83 ± 3	0.504 **<0.001**
Turn rate·s^−1^ (degrees) Males Turn rate·s^−1^ (degrees) Females	394 ± 9 349 ± 12	407 ± 11 317 ± 12	0.346 0.070
Turn bias·s^−1^ (degrees) Males Turn bias·s^−1^ (degrees) Females	−1.22 ± 0.98 −1.80 ± 1.16	−3.19 ± 1.30 −2.07 ± 1.17	0.231 0.873
Time in spawning area (s) Males Time in spawning area (s) Females	621 ± 30 426 ± 20	609 ± 44 382 ± 29	0.828 0.221
Path in spawning area M (m) Males Path in spawning area F (m) Females	67 ± 3 35 ± 2	64 ± 5 25 ± 2	0.596 **<0.001**
Visits to spawning area Males Visits to spawning area Females	642 ± 23 313 ± 17	626 ± 28 241 ± 16	0.653 **0.002**
Number of contacts between male and female	1309 ± 55	1081 ± 45	**0.002**
Total time (s) of contact between male and female	743 ± 42	787 ± 55	0.531
Average distance (mm) between male and female	73 ± 3	75 ± 3	0.717

All entries are average values ± SEM. Values that are significantly different (*p* < 0.05) from the control group are emphasized in bold. In the “Turn bias” row, negative values indicate a bias towards left turning.
